# Identification of mitophagy-related biomarkers in severe acute pancreatitis: integration of WGCNA, machine learning algorithms and scRNA-seq

**DOI:** 10.3389/fimmu.2025.1594085

**Published:** 2025-05-28

**Authors:** Xiaozhou Xie, Zheng Wang, Haoyu Zhang, Jiongdi Lu, Feng Cao, Fei Li

**Affiliations:** ^1^ Department of General Surgery, Xuanwu Hospital, Capital Medical University, Beijing, China; ^2^ Clinical Center for Acute Pancreatitis, Capital Medical University, Beijing, China

**Keywords:** severe acute pancreatitis, mitophagy, MAPK14, p38α, WGCNA, machine learning, immune cell infiltration, single-cell RNA sequencing

## Abstract

**Background:**

Mitophagy is a highly conserved cellular process in eukaryotic cells that selectively clears dysfunctional or damaged mitochondria through autophagy mechanisms to maintain mitochondrial homeostasis. However, the role of mitophagy in the pathogenesis of severe acute pancreatitis (SAP) has not been fully investigated. In this study, we aimed to identify crucial mitophagy-related genes in SAP to provide a theoretical basis for in-depth mechanistic investigations.

**Methods:**

We downloaded the GSE194331 dataset from the Gene Expression Omnibus (GEO), identified differentially expressed genes (DEGs), and used weighted gene co-expression network analysis (WGCNA) and three machine learning algorithms to identify crucial genes. In addition, single sample gene set enrichment analysis (ssGSEA) was conducted to explore the relationship between crucial genes and immune infiltration. The expression of crucial genes at the single-cell level was analyzed using single-cell RNA sequencing (scRNA seq) data from the GSE279876 dataset. Finally, we established the SAP mouse model and conducted preliminary validation of the mechanism of crucial genes in SAP.

**Result:**

We identified MAPK14 as a crucial mitophagy-related gene in SAP by intersecting the results of DEGs, WGCNA, and three machine learning algorithms. In addition, ssGSEA revealed that MAPK14 was strongly associated with immune cell infiltration. The analysis of scRNA-seq data revealed that MAPK14 was highly expressed in pancreatic macrophages, suggesting that macrophage-derived MAPK14 may potentially regulate inflammation in SAP. Finally, we preliminarily validated using the SAP mouse model that inhibiting the protein encoded by MAPK14 increased the expression of mitophagy marker proteins and significantly alleviated SAP inflammation.

**Conclusion:**

Inhibition of MAPK14 activation may alleviate SAP by enhancing mitophagy. Our study highlights the potential role of the mitophagy-related gene MAPK14 in SAP pathogenesis, providing important insights for future investigations into mitophagy-mediated immune mechanisms in SAP.

## 1 Introduction

Acute pancreatitis (AP) is a both localized and systemic inflammatory response triggered by pancreatic duct obstruction secondary to gallstones, long-term alcohol consumption, and other etiologies (1). AP has a variable course and unpredictable changes in the condition. Without timely diagnosis and treatment, approximately one-fifth of patients develop severe acute pancreatitis (SAP) (2). SAP is often characterized by pancreatic or peri-pancreatic tissue necrosis, systemic inflammatory response syndrome (SIRS), and persistent (>48h) single or multiple organ failure, with a mortality rate of approximately 20% (2–4). SAP patients exhibit two mortality peaks: the first associated with early organ failure within two weeks of onset and the second with sepsis due to infectious pancreatic necrosis (5). Given its numerous complications and high mortality rate, rigorous exploration of the molecular mechanisms underlying pancreatitis pathogenesis is critical for alleviating clinical symptoms and improving prognosis in SAP patients (6).

Autophagy is a process in which eukaryotic cells use lysosomes to degrade their own cytoplasmic proteins and damaged organelles under the regulation of autophagy-related genes (7). Based on the substrates encapsulated and their transport pathways to lysosomes, autophagy is categorized into macroautophagy, microautophagy, and chaperone-mediated autophagy (8). It can be further subdivided into nonselective and selective autophagy depending on whether the degraded substrate is specific (9). As a key form of selective autophagy, mitophagy is essential for maintaining cellular and mitochondrial homeostasis (10). Under external stimuli such as reactive oxygen species (ROS) stress, nutrient deficiency, or cellular aging, intracellular mitochondria undergo depolarization damage, losing their outer membrane potential. Subsequently, autophagosomes recognize and encapsulate damaged mitochondria, which then bind to lysosomes to promote the degradation of mitochondrial contents (11). Mitophagy plays a critical regulatory role in inflammatory diseases including acute lung injury (12), renal tubular inflammation (13), neuroinflammation (14), osteoarthritis (15), thyroiditis (16), and viral myocarditis (17). The PINK1/Parkin pathway has been found to alleviate AP by regulating mitophagy (18, 19). However, the molecular mechanism of mitophagy in SAP remain poorly understood and effective therapeutic targets are still lacking.

Advances in technologies such as bioinformatics and machine learning have allowed us to use a variety of tools for in-depth analysis of various diseases (20–22). We used three machine learning algorithms in this study. Specifically, the least absolute shrinkage and selection operator (LASSO) algorithm determines variables by finding the λ value with the smallest classification error, which is mainly used to screen feature variables and construct the optimal classification model (23). The random forest (RF) algorithm improves prediction accuracy by constructing multiple decision trees and combining their results to screening genes with the most significant impact on the phenotype (24). Support vector machine-recursive feature elimination (SVM-RFE), a feature selection method based on SVM, is commonly used to screen important features related to the target variable in high-dimensional data (24). Weighted gene co-expression network analysis (WGCNA) is a systems biology method used to describe gene association patterns between different samples, which can identify candidate biomarker genes or therapeutic targets based on expression correlations between gene sets and associations between gene sets and phenotypes (25). According to published literature, research on the role of mitophagy in SAP remains in its infancy. The unique pathological environment of SAP may lead to significantly different expression patterns and regulatory networks of mitophagy-related genes (MRGs) compared to those in other diseases. Therefore, further screening for potential genes that regulate mitophagy and inflammation in SAP is warranted.

In this paper, we integrated multiple approaches, including bioinformatics analyses, WGCNA, machine learning algorithms and single-cell RNA sequencing (scRNA-seq), to explore crucial biomarkers associated with mitophagy in SAP and screen mitogen-activated protein kinase 14 (MAPK14) as a potential therapeutic target for SAP. p38 kinase is a serine/threonine kinase of the mitogen-activated protein kinase (MAPK) family, and MAPK14 encodes the p38α protein, which is the best-characterized member of the p38 kinase family (26). Environmental stress, cytokines, and other pro-inflammatory factors can activate p38α and modulate immune and stress responses (27). Inhibition of p38 to promote mitophagy has been found to be therapeutic in Parkinson’s disease caused by dopaminergic neurodegeneration (28, 29). However, its role in regulating mitophagy in SAP remains unclear. We validated its expression in SAP mouse models through immunohistochemistry (IHC) experiments, and further demonstrated that inhibiting p38α with the specific inhibitor SB203580 upregulates mitophagy marker proteins and alleviates SAP. These findings provide a new perspective on the role of mitophagy in SAP pathogenesis and contributes to subsequent in-depth intervention studies.

## 2 Materials and methods

### 2.1 Data collection and processing

The datasets GSE194331 and GSE279876 were downloaded from the GEO database (https://www.ncbi.nlm.nih.gov/geo/, accessed on 15 October 2024). GSE194331 is an RNA-seq dataset of peripheral blood from 87 clinical AP patients (Mild = 57, Moderately-Severe = 20, Severe = 10). We extracted peripheral blood gene expression data from 32 healthy individuals and 10 SAP patients for analysis. GSE279876 is a scRNA-seq dataset of pancreatic tissue from mice with AP, comparing normal diet and high-fat diet conditions. AP was induced by intraperitoneal injection of caerulein (50 μg/kg), every hour for 12 consecutive times. Then, pancreatic tissues from each group were collected and subjected to 10x single-cell sequencing. We extracted the data of the normal control group and the acute pancreatitis group induced by a normal diet for analysis.

The GeneCards database (https://www.genecards.org/, accessed on 20 October 2024) was searched for “mitophagy” to obtain MRGs, and genes with relevance score >2 were selected. We search “mitophagy” in PubMed to obtain MRGs from the literature (30). After removing duplicates, 218 MRGs were finally obtained (**Supplementary Material 1**).

### 2.2 Identification of differentially expressed genes

The raw count data were normalized and then analyzed for differential expression using the “DESeq2” package in the R software. Genes with |logFC| > 1 and adjusted *p* < 0.05 were considered DEGs, and volcano and heat maps were plotted using the ggplot2 and “pheatmap” packages.

### 2.3 Functional enrichment analysis

Gene symbol conversion was performed using the “org.Hs.eg.db” and “org.Mm.eg.db” packages, Gene Ontology (GO) and Kyoto Encyclopedia of Genes and Genomes (KEGG) enrichment analyses were performed using the “clusterProfiler” package, and the results were visualized using the “ggplot2” package.

### 2.4 WGCNA

The “WGCNA” package was used to construct the gene co-expression matrix. Samples were first clustered and the optimal soft threshold (β) was determined to be 11. Using parameters minModuleSize = 30 and MergeCutHeight = 0.25, we constructed a scale-free co-expression network, thereby converting the adjacency matrix into a topological overlap matrix (TOM). We performed cluster analysis to identify gene modules, and constructed a dendrogram via hierarchical clustering to calculate the correlation between module eigengenes and disease phenotypes.

### 2.5 Machine learning algorithms screen for hub genes

To further screen for hub genes, three machine learning algorithms were used: LASSO logistic regression, RF, and SVM-RFE. Previous studies have demonstrated the effectiveness of these algorithms for gene screening (31). The LASSO regression model was built using the “glmnet” package, with the minimum λ value was selected as the optimal parameter for prediction. We constructed multiple decision trees using the “randomForest” package and aggregated their results to perform classification, regression, and feature selection. For the SVM-RFE algorithm, we used the “e1071” and “caret” packages, applying five-fold cross-validation to obtain the results. Finally, venn diagrams were plotted to show the intersection of the three algorithms.

### 2.6 MAPK14 expression and ROC assessment

The “ggpubr” package was used to visualize the expression level of MAPK14. Receiver operating characteristic (ROC) analysis was performed using “pROC” package to evaluate its diagnostic ability in SAP.

### 2.7 Single-gene GSEA

The GO, KEGG, REACTOME, and HALLMARK gene sets were downloaded from the MSigDB database (https://www.gsea-msigdb.org/gsea/msigdb, accessed on 30 October 2024). Single-gene GSEA was performed using the “clusterProfiler” package to explore the potential functions of MAPK14, with results visualized via the “enrichplot” package.

### 2.8 ssGSEA

Single sample gene set enrichment analysis (ssGSEA) was performed using the “GSVA” package. Immune cell infiltration and immune function activity for each sample were calculated based on sample gene expression. Correlations between immune cells abundance and immune pathways activity were calculated using the “corrplot” package. The “ggplot2” package was used to draw boxplots to compare the differences in immune cells infiltration and immune pathways activity between the SAP and control groups. A subset of MAPK14 expression was extracted from the original expression matrix, and the immune cell abundance matrix was combined with the target gene expression matrix. A lollipop plot was created using the “ggplot2” package to demonstrate correlation between MAPK14 expression and immune cells abundance as well as immune pathways activity.

### 2.9 scRNA-seq data preprocessing, dimensionality reduction, clustering and visualization

Quality control and preprocessing of data were performed using the “Seurat” package. The “PercentageEigenSet” function was used to remove mitochondrial genes (“^mt-”) and red blood cell genes (Hba1, Hba2, Hbb, Hbd, Hbe1, Hbg1, Hbg2, Hbm, Hbq1, and Hbz). Final cell quality control was carried out based on the following parameters: nFeature_RNA > 300 & nFeature_RNA < 7000 & nCount_RNA > 1000 & percent.mt < 20 & percent.HB < 1. The “NormalizeData” function was used for data normalization, the “FindVariableFeatures” function was used to find 3000 highly variable genes, and the “ScaleData” function was used to standardize the data. After PCA dimensionality reduction, the “Harmony” package was used for data integration. Then, the “FindNeighbors” and “FindClusters” functions were employed for clustering, and the “DoubletFinder” package was used to remove double cells. The results were visualized through t-distributed stochastic neighbor embedding (tSNE). By referring to the CellMarker2.0 (http://bio-bigdata.hrbmu.edu.cn/CellMarker/index.html, accessed on 10 November 2024), PanglaoDB (https://panglaodb.se/index.html, accessed on 10 November 2024), and Cell Taxonomy (https://ngdc.cncb.ac.cn/celltaxonomy/, accessed on 10 November 2024) databases for cell annotation, 20643 cells were annotated as 11 cell types, including acinar cells, fibroblasts, neutrophils, macrophages, T cells, B cells, endothelial cells, mesothelial cells, beta cells, alpha cells, and pericytes.

### 2.10 Differential analysis of scRNA-seq data

The cluster-specific genes of macrophages were identified using the “FindAllMarkers” function. Subsequently, adjusted *p*-values were calculated using the Wilcoxon rank sum test. These differential genes were then used for GO and KEGG enrichment analysis.

### 2.11 SAP animals model construction

C57BL/6 mice were purchased from Beijing Vital River Laboratory Animal Technology Co., Ltd. Newly arrived mice were allowed free access to food and water for one week. Then they were fasted 12 h before the experiment. To induce SAP, mice were intraperitoneally injected with cerulein (MCE, China) at a dose of 100 μg/kg every 1 h for a total of 8 times. The last injection of cerulein was accompanied by 15 mg/kg of Lipopolysaccharide (MCE, China). For the SB203580 + SAP group, mice were intraperitoneally injected with SB203580 (10 mg/kg) 30 min before the first injection of cerulein. After the treatment, the mice were sacrificed by cervical dislocation, and pancreatic tissues were collected for subsequent experiments. This study was approved by the Ethics Committee of Xuanwu Hospital of Capital Medical University (XW20211223-1).

### 2.12 Hematoxylin-eosin staining

The tissue sections were deparaffinized in xylene and then hydrated through a series of alcohols with decreasing concentrations to facilitate dye penetration. They were stained with hematoxylin, rinsed under running water to remove excess dye, and then differentiated with 0.1% hydrochloric acid in ethanol. After another rinse to remove the acid, the sections were stained with eosin, followed by a further rinse under running water to remove excess eosin. Subsequently, the sections were dehydrated using a series of alcohols with increasing concentrations, mounted with a neutral resin, and photographed for observation. Two pathologists independently scored the severity of pancreatitis in mice according to Kusske et al.’s pancreatic pathology scoring criteria (32).

### 2.13 IHC staining

The tissue sections were placed in a thermostat at 60°C and baked for 2h. they were dewaxed in xylene and hydrated in gradient ethanol. Subsequently, antigen retrieval was performed using sodium citrate. The sections were then blocked with 5% goat serum. After blocking, they were incubated overnight at 4°C with primary antibodies against p38 MAPK (ABclonal, A14401) and phospho-p38 MAPK (ABclonal, AP0057). After the incubation with primary antibodies, the sections were incubated with secondary antibodies. Subsequently, the expression of target proteins was detected using 3,3′-diaminobenzidine (DAB) solution. Two professional pathologists independently scored the results using the immunoreactivity score (IRS) as follows: The intensity of cellular staining was divided into 4 levels, no positive staining (negative) scored 0, yellow (weakly positive) scored 1, tawny (positive) scored 2, and brown (strongly positive) scored 3. The percentage of positive cells was divided into 4 levels, ≤25% scored 1, 26%-50% scored 2, 51%-75% scored 3, >75% scored 4. The final score was obtained by multiplying these two scores.

### 2.14 Western blot

The pancreatic tissue was lysed on ice using RIPA lysis buffer and the supernatant was collected by centrifugation. Proteins were separated via sodium dodecyl sulfate-polyacrylamide gel electrophoresis (SDS-PAGE) and transferred to polyvinylidene fluoride (PVDF) membranes. After blocking the PVDF membranes with 5% skim milk, the membranes were incubated overnight at 4°C with primary antibodies targeting β-actin (Proteintech, 20536-1-AP), Pink1 (Proteintech, 23274-1-AP), Parkin (Proteintech, 14060-1-AP), and Bnip3l/Nix (Selleck, F0469). Then, the membranes were incubated with secondary antibodies at room temperature for 1 h. Finally, protein bands were visualized using a western blot imaging system.

### 2.15 Statistical analysis

Bioinformatics-related statistical analyses were performed using R software (R-4.4.1, 64-bit). For molecular biology experiments, data from at least three independent experimental replicates were analyzed using two-sample t-tests or Wilcoxon rank-sum tests using Prism 8 (GraphPad), A *p*-value < 0.05 was considered statistically significant.

## 3 Results

### 3.1 Identification of DEGs and functional enrichment analysis

**Figure 1:**
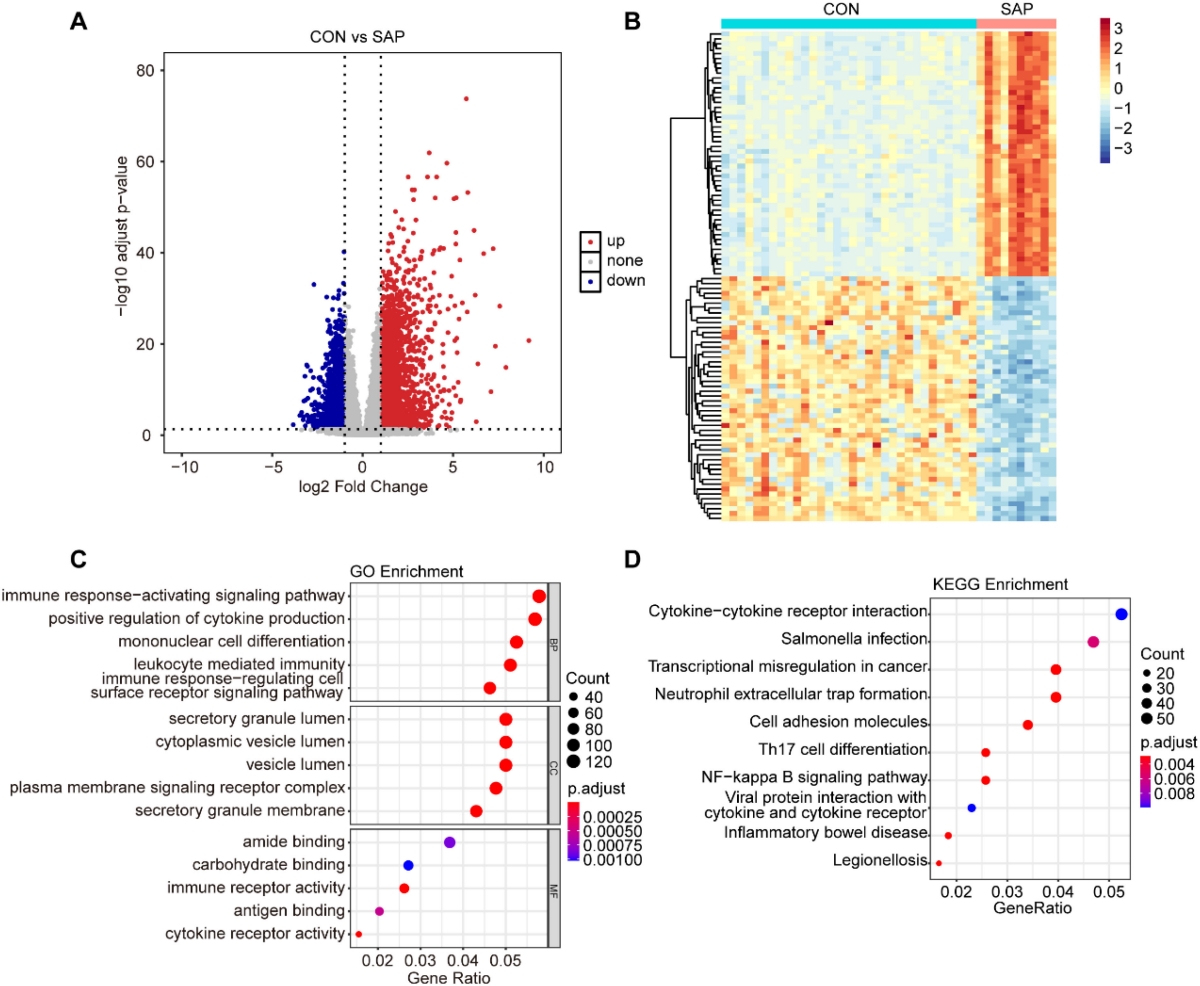
Identification and enrichment analysis of DEGs. **(A)** Volcano plot of DEGs between the SAP and control groups (cut-off criteria: |logFC| >1 and adjusted *p*-value < 0.05). **(B)** Heatmap of top50 DEGs (upregulated and downregulated) between the SAP and control samples. **(C)** GO enrichment analysis for DEGs. **(D)** KEGG enrichment analysis for DEGs.

In this study, we used the GSE194331 dataset, which included sample data from 10 SAP patients and 32 healthy individuals. DEGs were screened based on the criteria of |logFC|> 1 and an adjusted *p*-value < 0.05. A total of 4110 DEGs were identified, comprising 2290 up-regulated genes and 1820 down-regulated genes (**Figure 1A**). The top 50 up-regulated and top 50 down-regulated genes were respectively selected for heatmap visualization (**Figure 1B**).

To further explore the potential biological functions regulated by these DEGs, we conducted GO and KEGG enrichment analyses on the screened DEGs. The results indicated that these DEGs were primarily involved in various biological processes, including the immune response-activating signaling pathway, positive regulation of cytokine production, mononuclear cell differentiation, leukocyte mediated immunity, and immune response-regulating cell surface receptor signaling pathway. Moreover, the DEGs were also involved in multiple signaling pathways, such as cytokine-cytokine receptor interaction, Neutrophil extracellular trap formation, and NF-kappa B signaling pathway (**Figures 1C, D**).

### 3.2 Construction of co-expression network and screening of core genes

**Figure 2:**
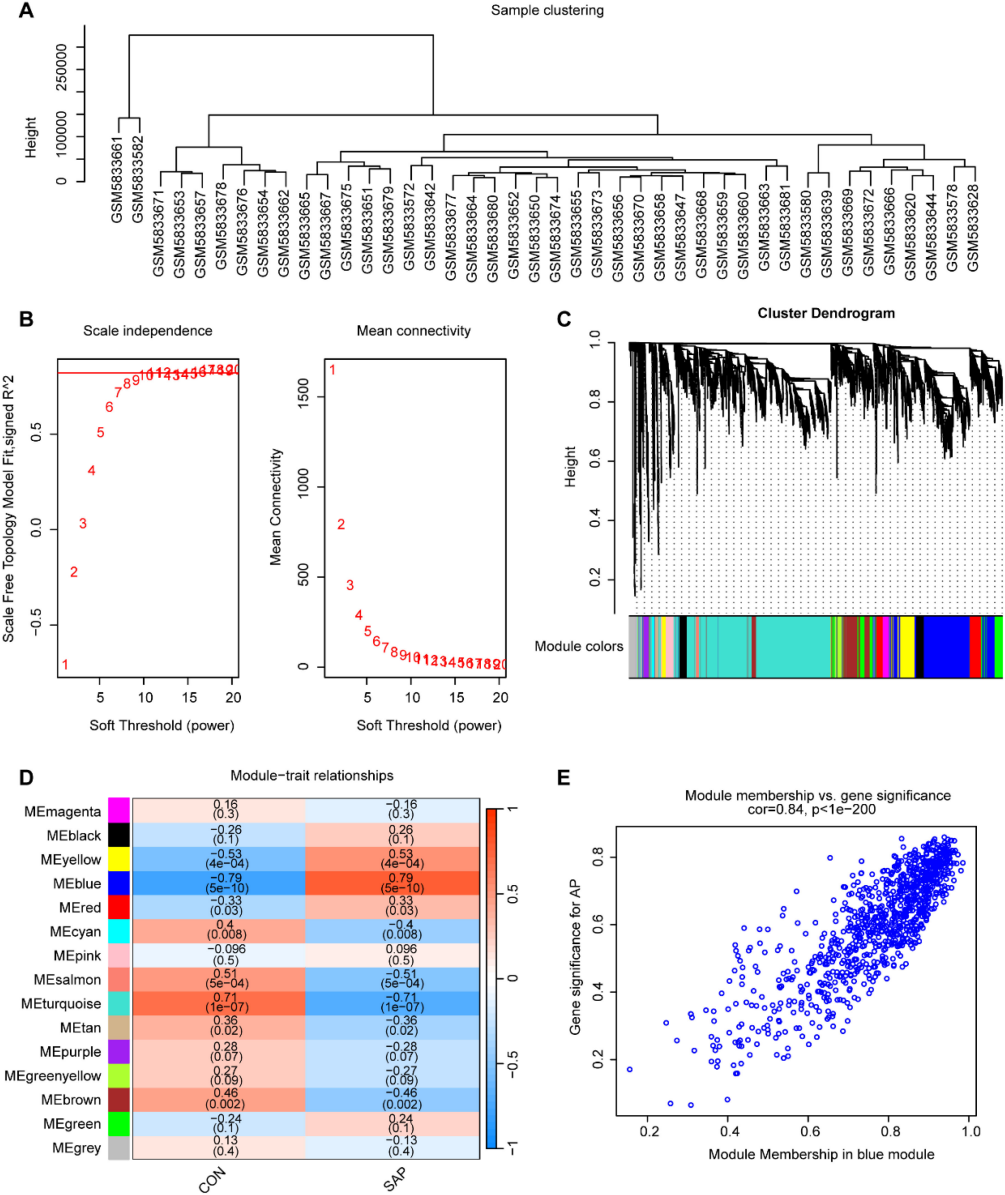
Construction of the co-expression network. **(A)** Sample clustering using average clustering methods. **(B)** Selection of a suitable soft threshold (power = 11) and scale-free topology fit index (R^2^ = 0.85). **(C)** Gene hierarchical clustering diagram. **(D)** Heatmap of correlations between gene modules and SAP. **(E)** Scatterplot between the GS and MM in the “blue” module.

To identify core genes associated with SAP, we constructed a gene co-expression network using WGCNA. Sample clustering analysis revealed distinct clustering of samples (**Figure 2A**), indicating reliable data quality. A soft threshold of β = 11 was selected, yielding a scale-free topology fit index (R²) of 0.85 (**Figure 2B**), which confirmed the construction of a scale-free co-expression network. Using gene correlation matrices, we constructed a hierarchical clustering dendrogram of genes, identifying 15 distinct gene modules (**Figure 2C**). Among these, the “blue” module, containing 924 genes, exhibited the strongest correlation with SAP phenotypes (Cor = 0.79, *p* = 5 × 10^-10^), making it the most clinically relevant module (**Figure 2D**). A scatter plot revealed a significant positive correlation between gene salience (GS) and module members (MM) within the “blue” module (Cor = 0.84, *p* < 1 × 10^-200^), indicating high consistency between module genes and SAP relevance (**Figure 2E**).

### 3.3 Selection and functional enrichment analysis of MRGs in SAP

**Figure 3:**
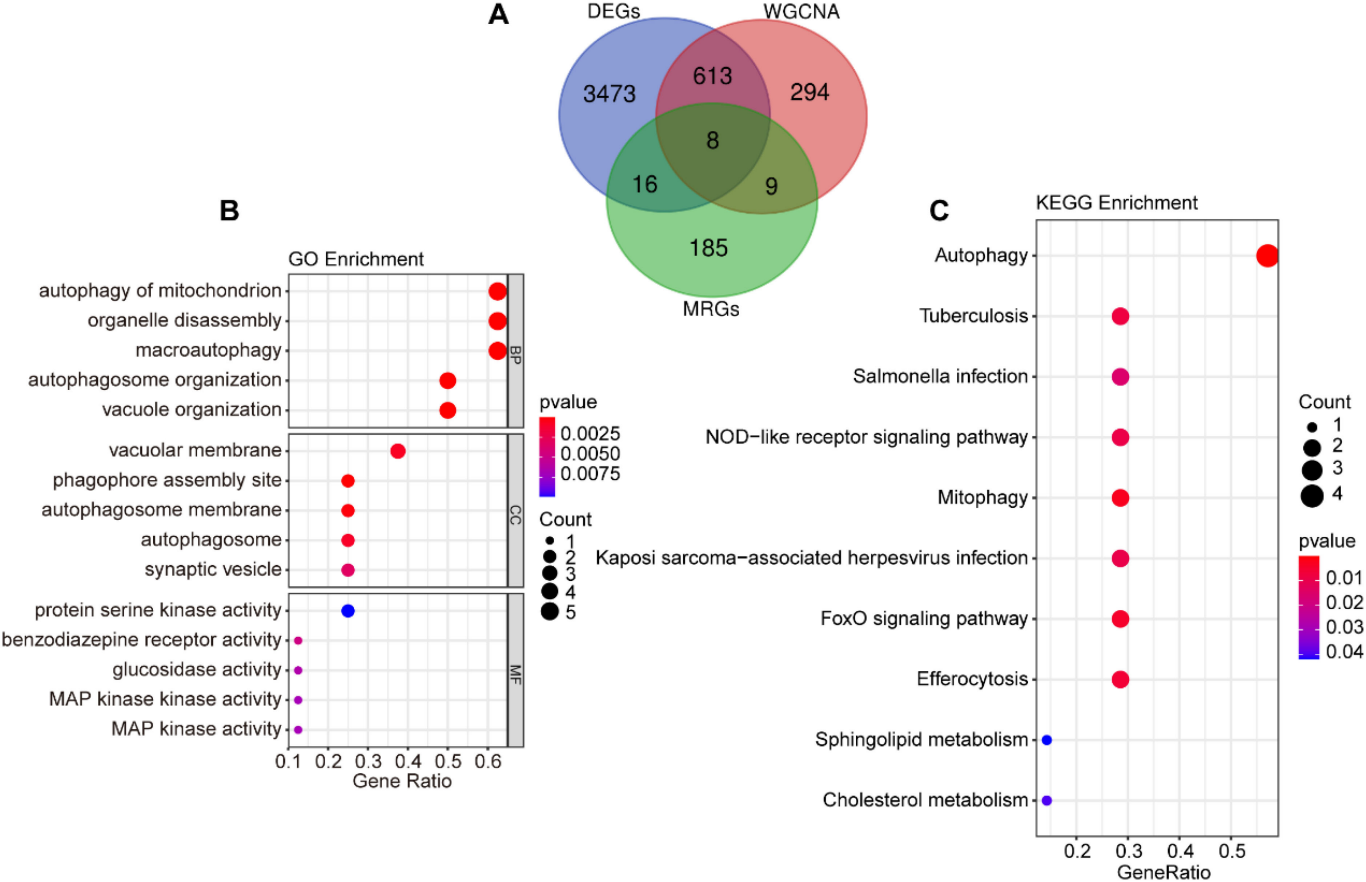
Screening of mitophagy-related signature genes. **(A)** Venn diagram of the intersection of DEGs, “blue” module genes, and MRGs. **(B)** GO enrichment analysis for the intersecting genes. **(C)** KEGG enrichment analysis for the intersecting genes.

To investigate the role of mitophagy in SAP pathogenesis, we performed an intersection analysis among DEGs, MRGs, and genes in the “blue” module, identifying 8 overlapping signature genes (**Figure 3A**). These genes were subjected to GO and KEGG enrichment analyses to characterize their potential biological functions and pathways associated with mitophagy in SAP pathogenesis. GO functional enrichment revealed that the signature genes mainly regulate biological functions such as autophagy of mitochondrion, organelle disassembly, macroautophagy, autophagosome organization, and vacuole organization (**Figure 3B**). KEGG pathway enrichment revealed their involvement in autophagy, NOD-like receptor signaling pathway, mitophagy, FoxO signaling pathway, efferocytosis, sphingolipid metabolism, and cholesterol metabolism (**Figure 3C**).

### 3.4 Machine learning algorithms identify core genes

**Figure 4:**
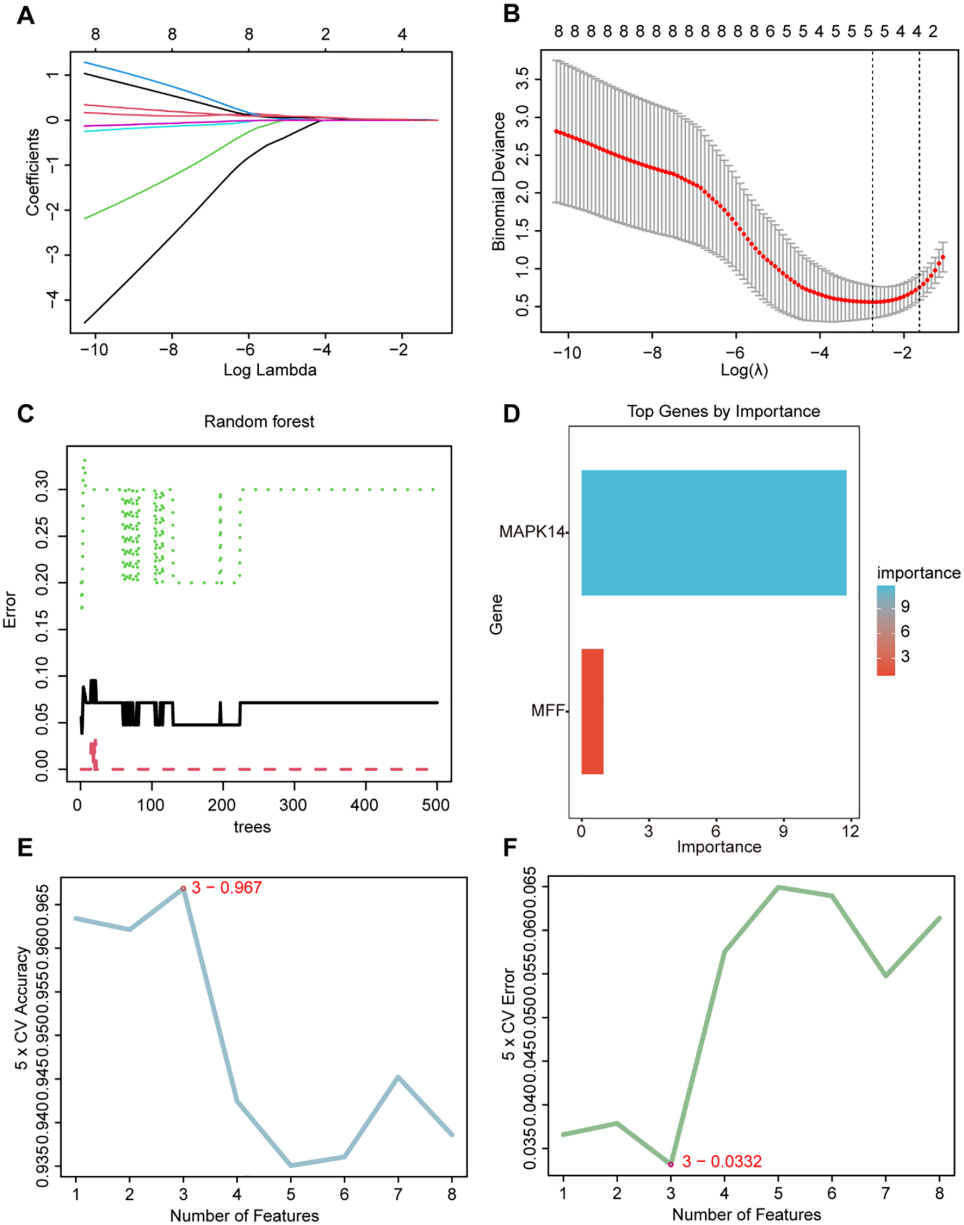
Identification of critical gene using three machine learning algorithms. **(A)** Path diagram for LASSO regression analysis with candidate MRGs. **(B)** LASSO regression cross-validation curves. A 10-fold cross-validation was used to determine the optimal λ value, and the optimal λ yielded 5 MRGs. **(C)** Correlation between the number of random forest trees and model errors. **(D)** RF importance score results (only MAPK14 and MFF received importance score). **(E)** Accuracy plot of the SVM-RFE algorithm. **(F)** Error plot of the SVM-RFE algorithm.

**Figure 5:**
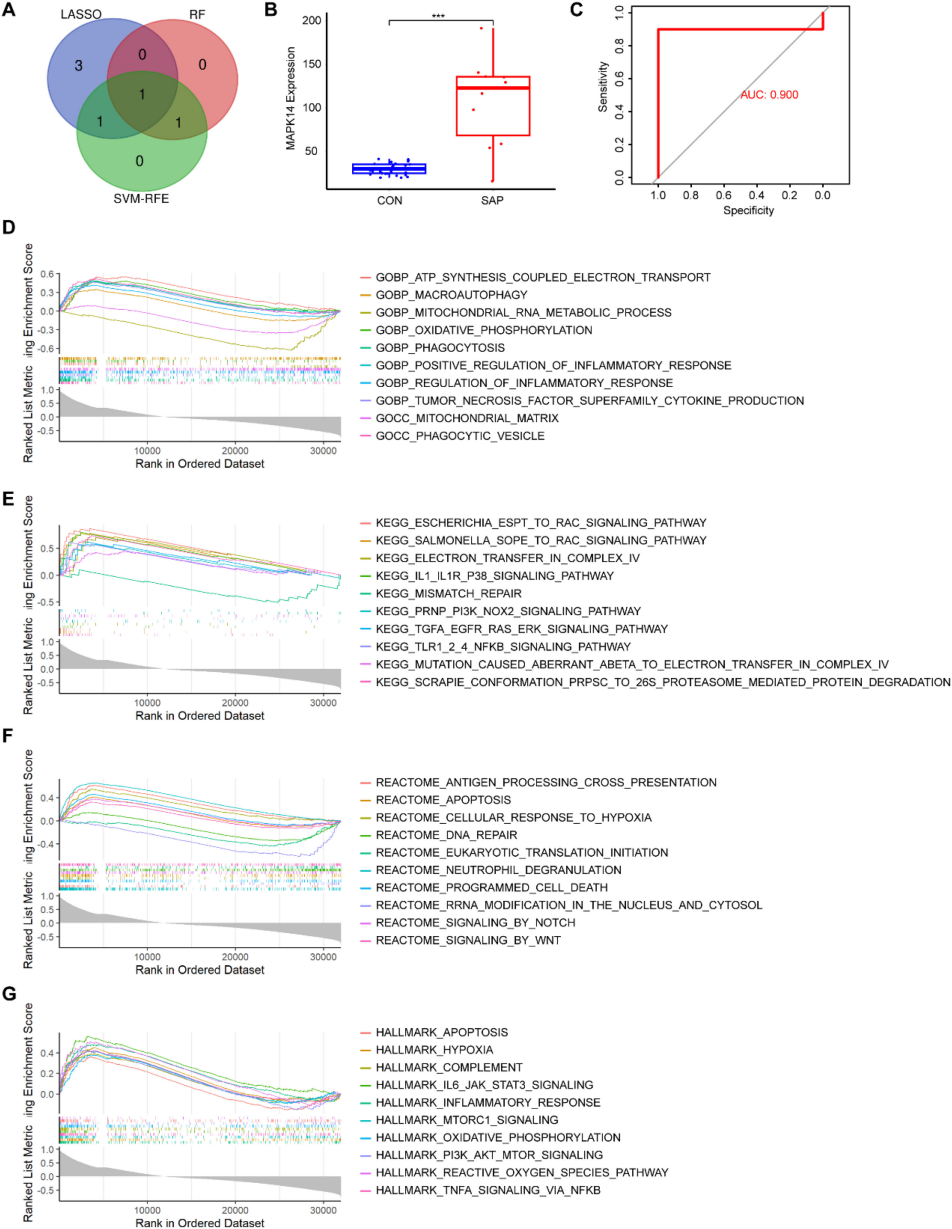
Diagnostic value and GSEA of MAPK14. **(A)** V Venn diagram showing the intersection of the results from three machine learning algorithms. **(B)** Expression levels of MAPK14 in SAP and control samples. **(C)** Receiver operating characteristic (ROC) curves for evaluating the diagnostic value of MAPK14. **(D)** GSEA of MAPK14 in the GO dataset. **(E)** GSEA of MAPK14 in the KEGG dataset. **(F)** GSEA of MAPK14 in the REACTOME dataset. **(G)** GSEA of MAPK14 in the HALLMARK dataset. *** p < 0.001 represents significance.

We utilized three machine learning algorithms to further screen for core genes. Using LASSO regression, we identified five core genes that best characterize the MRGs in SAP: ATG3, MAPK14, CAMKK2, TSPO, and GABARAPL2 (**Figures 4A, B**). RF identified two significant genes: MAPK14 and MFF (**Figures 4C, D**), SVM-RFE identified three candidate genes with minimal error and maximal accuracy: MAPK14, MFF, and ATG3 (**Figures 4E, F**). By intersecting the results from the three algorithms, we identified MAPK14 as the mitophagy-related gene most strongly associated with SAP (**Figure 5A**). Further analysis revealed that MAPK14 expression was significantly higher in SAP patients than in healthy individuals (**Figure 5B**). Moreover, ROC curve analysis showed that MAPK14 had a good predictive value for SAP, with an AUC value of 0.900 (**Figure 5C**).

### 3.5 Single-gene GSEA of MAPK14

Our previous analysis identified MAPK14 as the mitophagy-related gene most strongly associated with SAP pathogenesis, potentially regulatory SAP progression. To further explore its molecular functions and regulated biological pathways, we performed single-gene GSEA. MAPK14 was enriched in GO terms including ATP synthesis coupled electron transport, macroautophagy, mitochondrial RNA metabolic process, oxidative phosphorylation, and positive regulation of inflammatory response (**Figure 5D**). GSEA of the KEGG gene set revealed that MAPK14 is involved in regulating pathways such as electron transfer in complex IV, IL1-IL1R-p38 signaling pathway, mismatch repair, PRNP-PI3K-NOX2 signaling pathway, and TGFR-EGFR-RAS-ERK signaling pathway (**Figure 5E**). GSEA of the REACTOME gene set revealed that MAPK14 is involved in regulating pathways such as antigen processing cross presentation, apoptosis, cellular response to hypoxia, DNA repair, and eukaryotic translation initiation (**Figure 5F**). And GSEA of the HALLMARK gene set revealed that MAPK14 is involved in regulating pathways such as apoptosis, hypoxia, complement, IL6-JAK-STAT3 signaling, and inflammatory response (**Figure 5G**). This further highlights the potential of targeting MAPK14 to modulate SAP acinar cell death and inflammation.

### 3.6 Immune cell infiltration and functions and its association with MAPK14

**Figure 6:**
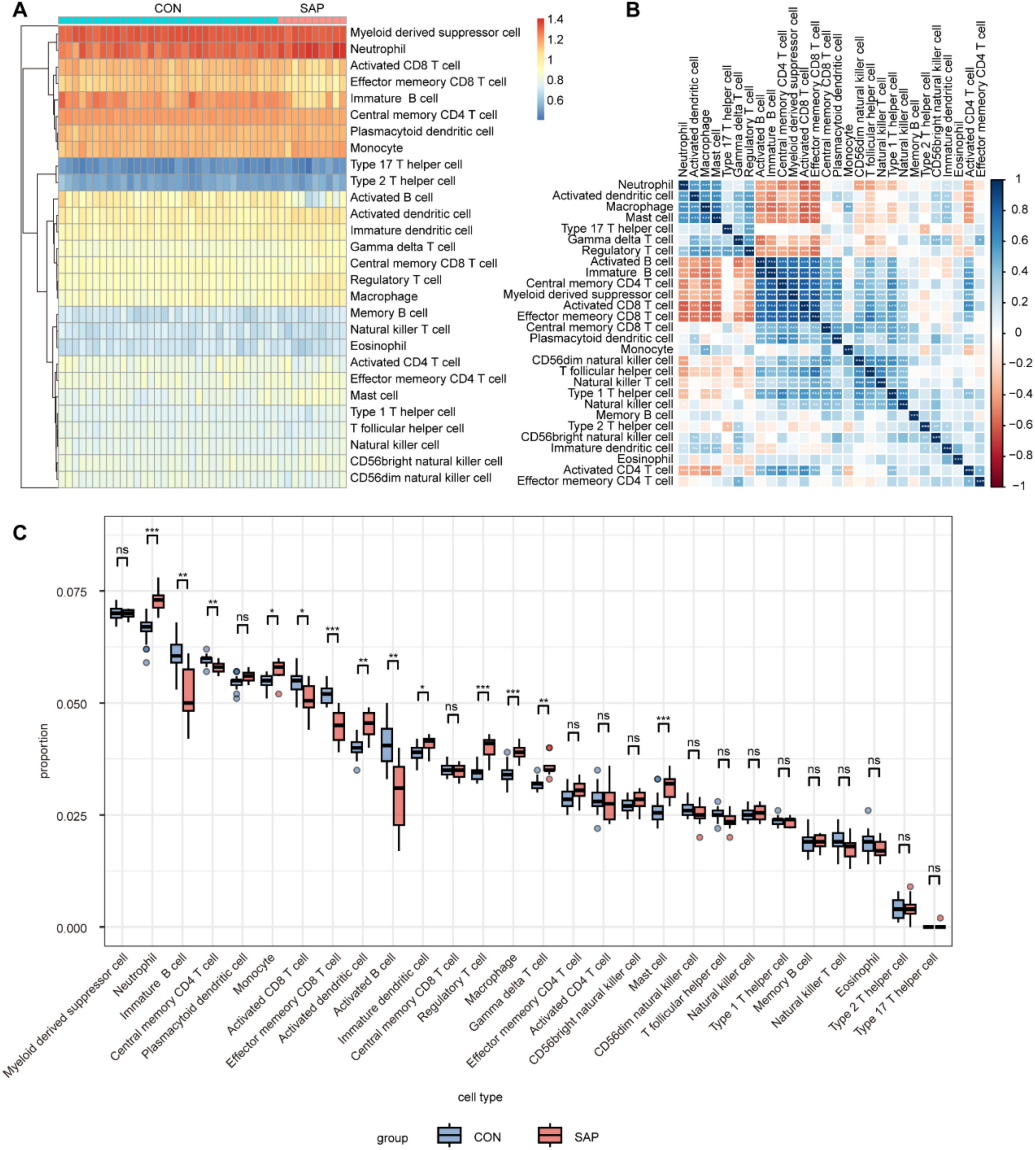
Analysis of immune cell infiltration in SAP and control samples using ssGSEA. **(A)** Heatmap of 28 immune cell types in SAP and control samples. **(B)** Heatmap of correlation between 28 immune cell types. **(C)** Boxplots showing infiltration scores of 28 immune cell types in SAP and control samples. *p < 0.05, **p < 0.01, and ***p < 0.001 represent varying degrees of significance between the indicated groups.

**Figure 7:**
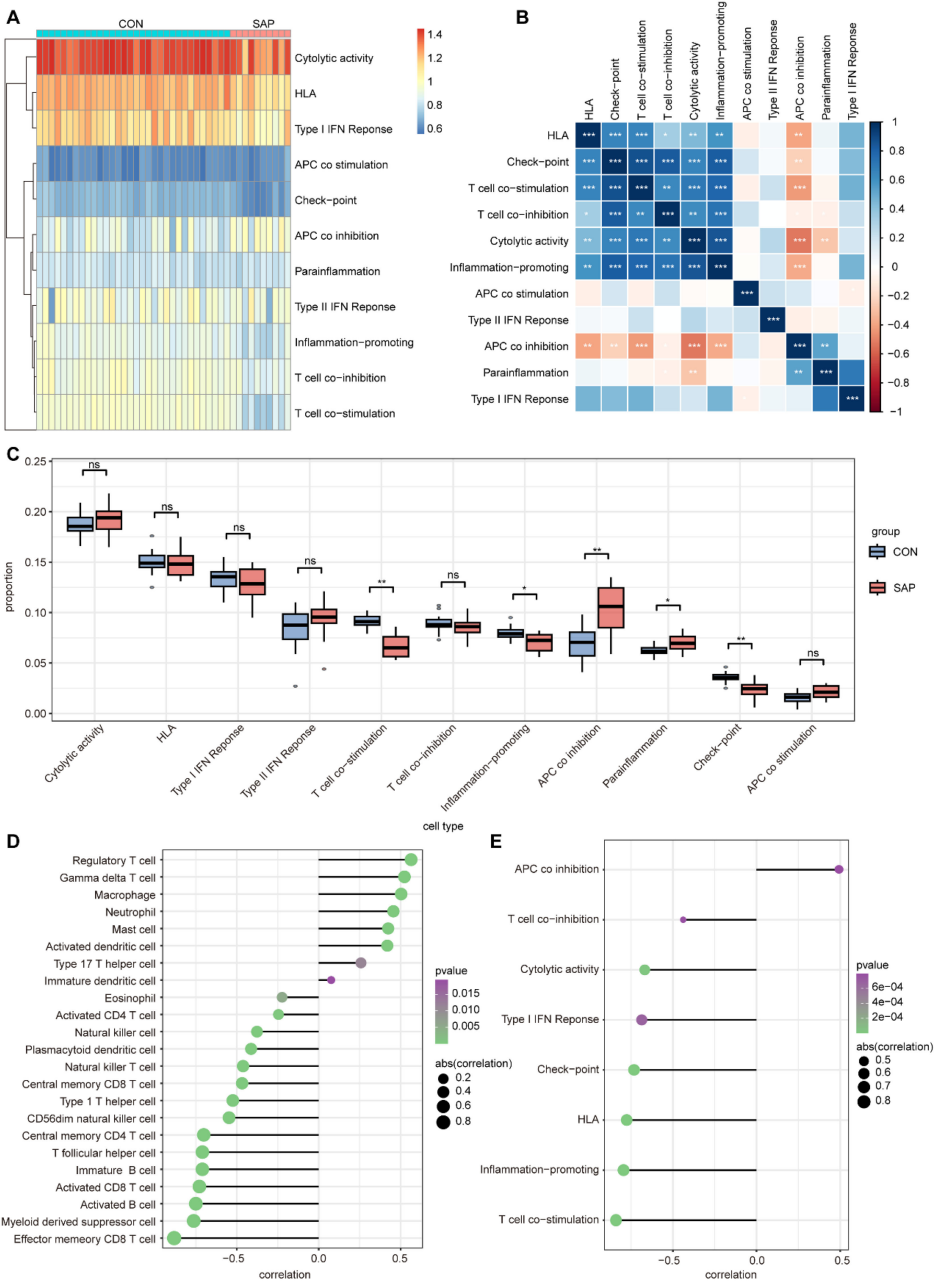
Analysis of immune function in SAP and control samples using ssGSEA. **(A)** Heatmap for 11 immune functions in SAP and control samples. **(B)** Heatmap of correlation between the 11 immune functions. **(C)** Boxplots showing scores of 11 immune function in SAP and control samples. **(D)** Lollipop plot depicting the correlation between MAPK14 and immune cell infiltration. **(E)** Lollipop plot depicting the correlation between MAPK14 and immune functions. *p < 0.05, **p < 0.01, and ***p < 0.001 represent varying degrees of significance between the indicated groups.

Immune mechanisms play an important role in the development of SAP. Therefore, we explored the differences in immune infiltration between SAP patients and normal individuals using ssGSEA. The heatmap shows the distribution of 28 immune cells in 32 healthy individuals and 10 SAP patients in the GSE194331 dataset (**Figure 6A**). **Figure 6B** shows the correlation between these 28 immune cells. We observed that the infiltration of neutrophil, monocyte, activated dendritic cell, immature dendritic cell, regulatory T cell, macrophage, gamma delta T cell, and mast cell was significantly higher in SAP, while the infiltration of immature B cell, central memory CD4 T cell, activated CD8 T cell, effector memory CD8 T cell, and activated B cell decreased in SAP. This suggesting that these immune cells may play a crucial role in the development of SAP (**Figure 6C**). In addition, we further explored the distribution of 11 immune functions between SAP patients and healthy individuals, as well as the correlation between these 11 immune functions (**Figures 7A, B**). Differential analysis of immune function revealed that T cell co-stimulation, inflammation-promoting, APC co inhibition, parainflammation, and check-point were closely associated with the development of SAP (**Figure 7C**). Correlation analysis revealed that MAPK14 was statistically correlated with 23 immune cells and 8 immune functions. Among them, regulatory T cell, gamma delta T cell, macrophage, neutrophil, mast cell, activated dendritic cell, type 17 T helper cell, immature dendritic cell, and APC co inhibition immune function were positively correlated with MAPK14 (**Figures 7D, E**).

### 3.7 scRNA-seq analysis of MAPK14

**Figure 8:**
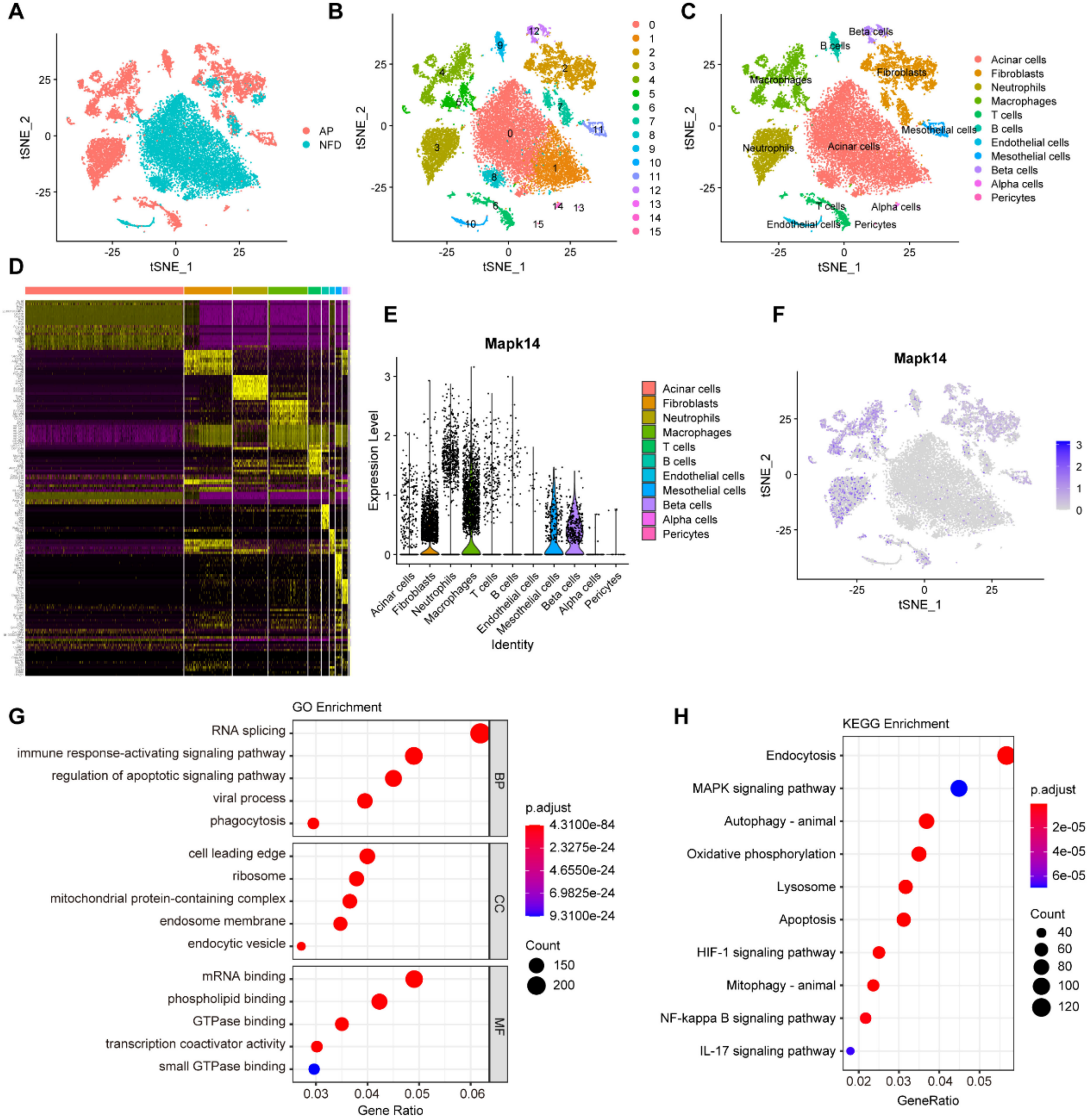
Single-cell transcriptome analysis of pancreatic cells. **(A)** Visualization of tSNE for AP and control samples. **(B)** tSNE visualization of 15 cell clusters. **(C)** tSNE visualization of acinar cells, fibroblasts, neutrophils, macrophages, T cells, B cells, endothelial cells, mesothelial cells, beta cells, alpha cells, and pericytes. **(D)** Marker genes for the 15 cell clusters. **(E)** Violin plot showing the expression of Mapk14 across all cell types. **(F)** tSNE visualization depicting the expression of Mapk14. **(G)** GO enrichment analysis of differential expressed genes in macrophage subpopulations. **(H)** KEGG pathway enrichment analysis of differential expressed genes in macrophage subpopulations.

To investigate the potential role of MAPK14 in AP at the single-cell level, we analyzed the scRNA-seq data from the NFD group (control) and the AP group in the GSE279876 dataset. A total of 20643 cells from C57BL/6 mouse pancreatic samples were processed, and the dataset underwent dimensionality reduction, clustering, and tSNE visualization (**Figure 8A**). Fifteen major cell clusters were identified, and the “FindAllMarker” package was used to identify marker genes (**Figure 8B**). Based on these markers, 20643 cells were annotated into 11 cell types: acinar cells, fibroblasts, neutrophils, macrophages, T cells, B cells, endothelial cells, mesothelial cells, beta cells, alpha cells, and pericytes (**Figure 8C**). **Figure 8D** displays the marker genes for the 15 cell clusters. We found that Mapk14 was expressed across multiple cell types, with the highest expression observed in macrophages (**Figures 8E, F**). To explore its role in macrophages, we extracted macrophage subpopulations for differential gene expression analysis and performed GO and KEGG enrichment analysis. Differentially expressed genes were primarily involved in biological processes such as RNA splicing, immune response activating signaling pathway, and regulation of apoptotic signaling pathway (**Figure 8G**), as well as pathways such as endocytosis, MAPK signaling pathway, autophagy, oxidative phosphorylation, lysosome, and apoptosis (**Figure 8H**). These findings indicate that Mapk14 may play a regulatory role in these biological processes, highlighting its potential importance in macrophage-mediated functions during AP.

### 3.8 Inhibition of p38α relieves SAP

**Figure 9:**
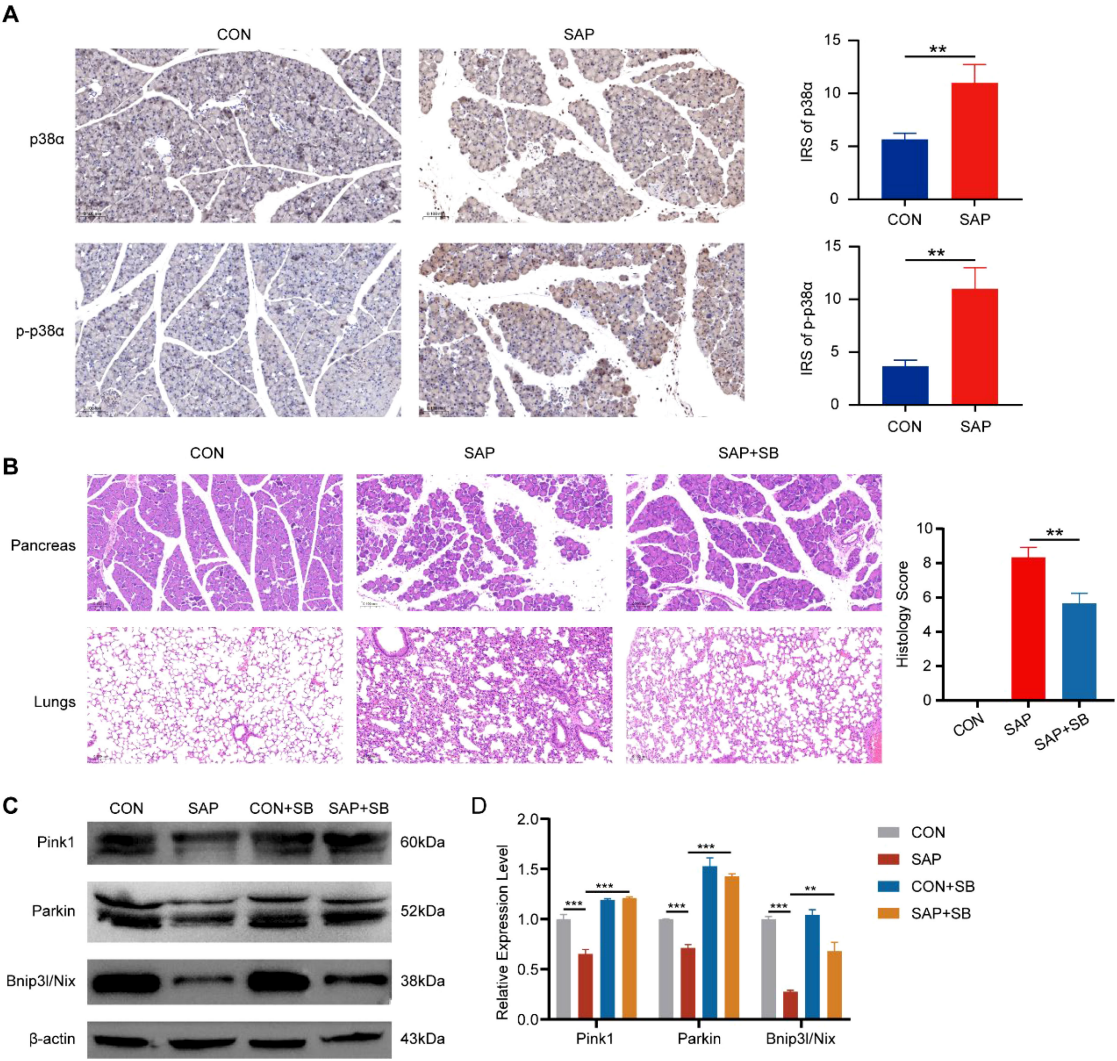
Effects of p38α inhibition in SAP mice. **(A)** IHC staining for p38α and phosphorylated p38α in pancreatic tissues of SAP and control mice (magnification, 200×). **(B)** HE staining showing the effect of the p38α inhibitor SB203580 (10 mg/kg) on pancreatic inflammation and lung injury (magnification, 200×). **(C)** Western blot showing the effect of p38α inhibition on the expression of mitophagy marker proteins. **(D)** Band density was measured (ImageJ software) and normalized to that of β-actin. ** p < 0.01, and *** p < 0.001 represent varying degrees of significance between the indicated groups.

To investigate the role of p38α in SAP pathogenesis *in vivo*, we established SAP mouse models. IHC staining of mouse pancreatic tissues revealed that p38α expression was significantly higher in SAP mice than in control mice, with phosphorylated p38α exhibiting a more pronounced increase (**Figure 9A**). This indicates activation of the p38α in the pancreas of SAP mice. To validate the role of p38α in SAP-associated inflammation, we treated mice with the p38-specific inhibitor SB203580 and observed significant reductions in pancreatic inflammation and lung injury (**Figure 9B**). In addition, we found that the expression of mitophagy markers Pink1, Parkin, and Bnip3l/Nix was reduced in the pancreatic tissue of SAP mice, indicating impaired mitophagy in SAP (**Figures 9C, D**). The expression of these markers was restored after p38α inhibition (**Figures 9C, D**). This suggests that inhibiting p38α may alleviate pancreatitis severity by promoting mitophagy in SAP.

## 4 Discussion

SAP is a complex and rapidly progressive form of severe pancreatic inflammation, in which uncontrolled inflammatory responses lead to pancreatic necrosis and multiorgan failure, thereby inducing severe complications such as acute respiratory distress syndrome (ARDS) and shock (33). Despite significant progress in the treatment of SAP in recent years, clarifying the molecular mechanisms underlying its pathogenesis and progression remains a pressing issue.

Mitophagy selectively eliminates dysfunctional or damaged mitochondria to maintain normal mitochondrial function and prevent inflammation caused by mitochondrial damage (34). The normal function of pancreatic acinar cell organelles, including mitochondria, is essential for maintaining pancreatic physiology. Previous studies have revealed mitochondrial swelling, cristae disruption, and dysfunction in AP (35), which can lead to consequences such as pancreatic endoplasmic reticulum stress, impaired autophagy, and dysregulation of lipid metabolism, which in turn exacerbate AP (36). Available studies suggest that mitophagy regulates AP severity through two major pathways: ubiquitin-mediated and receptor-mediated (10). Ubiquitin mediated mitophagy is executed via the PINK1/Parkin pathway. PINK1, a serine/threonine protein kinase, eliminates damaged mitochondria by activating the ubiquitin E3 ligase Parkin (37, 38). Receptor-mediated mitophagy relies on outer mitochondrial membrane receptors (e.g., BNIP3L/NIX), which initiate mitophagy by directly interacting with LC3 on autophagosome membranes (39). Recent studies highlight that mitochondrial dysfunction and impaired or deficient mitophagy are critical mechanisms in AP/SAP pathogenesis (10). For example, PINK1/Parkin-dependent mitophagy attenuates AP by inhibiting NLRP3 (18), while MRG15 promotes apoptosis by suppressing mitophagy in hyperlipidemic AP (40). Sestrin2 attenuates SAP by balancing mitophagy and apoptosis through the PINK1/Parkin pathway (19).

In this study, we integrated various bioinformatics tools to identify MAPK14 as a key mitophagy-related biomarker in SAP. To characterize the biological roles of MAPK14, we performed single-gene GSEA across four gene sets (GO, KEGG, REACTOME, and HALLMARK). Results showed that MAPK14 may regulate mitochondrial electron transport and RNA metabolism, in addition to modulating mitophagy and inflammatory responses. and furthermore, It was also involved in pathways such as apoptosis and IL6-JAK-STAT3, suggesting potential mechanisms for its role in mitophagy regulation. Immune cell infiltration is critical in SAP pathogenesis (41). Using ssGSEA, we analyzed the correlation between MAPK14 and immune cell infiltration/functions. Compared with healthy controls, we observed increased infiltration of immune cells in SAP patients, including neutrophil, monocyte, activated dendritic cell, immature dendritic cell, regulatory T cell, macrophage, gamma delta T cell, and mast cell. Notably, MAPK14 was positively correlated with predominantly infiltrating immune cells in SAP, such as neutrophils and macrophages, indicating that infiltration of these cell types may be associated with MAPK14 activation in SAP. Our further analysis using scRNA-seq datasets of mouse pancreatic tissues revealed predominant Mapk14 expression in pancreatic macrophages, suggesting that macrophage-derived Mapk14 plays a key role in regulating AP inflammation.

The p38 kinase family comprises four isoforms: p38α, p38β, p38γ, and p38δ, with p38α (encoded by MAPK14) being the most abundant subtype (42). As a critical immune-inflammatory regulator, p38α is activated by oxidative stress, ischemia, hypoxia, and endotoxin, promoting the release of pro-inflammatory cytokines, such as IL-1β, TNF-α, and IL-6, and exacerbating inflammatory responses (43). While p38-mediated mitophagy regulation has been preliminarily demonstrated in neurological disorders. For example, p38 inhibition mitigates Park2 deficiency-induced ROS generation and mitochondrial dysfunction in Parkinson’s disease (29). Subsequently, p38 was found to negatively regulate Parkin, and p38 inhibition prevented progressive neuronal degeneration by enhancing mitophagy (28). Its role in SAP remains unclear. Previous studies reported that p38α regulates SAP via macroautophagy (44), but whether it participates in mitochondrial quality control through selective mitophagy to modulate SAP inflammation is unknown. We found that p38α was activated in SAP tissues and observed significant alleviation of pancreatic inflammation and lung injury following p38α inhibition, providing preliminary evidence for its therapeutic efficacy in SAP. Further, we found that the expression of mitophagy regulatory proteins Pink1, Parkin, and Bnip3l/Nix was significantly reduced in SAP, and their expression was restored after inhibiting p38α, indicating that p38α inhibition alleviates SAP by enhancing mitophagy.

In summary, targeting p38α significantly relieved SAP in mice, highlighting its clinical potential as a therapeutic target. In addition, the data for GSE194331 used in this study came from peripheral blood of clinical patients. The expression of MAPK14 is significantly elevated in SAP patients, and according to the ROC curve, MAPK14 has great predictive value for SAP, highlighting its value in the diagnosis of SAP. However, limitations exist: first, our analysis relied solely on the GEO database due to the scarcity of non-oncology databases, lacking validation with our own sequencing data; second, due to the difficulty of clinical sampling of tissues from SAP patients, we could only utilize peripheral blood sample RNA-seq data from SAP patients for our analysis; third, the current public database only contains scRNA-seq data from AP mouse models, and the lack of corresponding scRNA-seq data from SAP mouse models precluded single-cell level analysis of SAP pathogenesis; finally, while we demonstrated that p38α inhibition enhances mitophagy marker proteins expression and alleviates inflammation in SAP mice, the specific molecular mechanisms require further investigation.

## 5 Conclusions

In conclusion, through comprehensive bioinformatics analysis, we identified and validated MAPK14 as a critical mitophagy-related gene in SAP. Our findings preliminarily demonstrated that p38α inhibition upregulates the expression of mitophagy marker proteins and effectively alleviated SAP. These results provide a research direction and foundation for further investigating the regulation of SAP inflammation through mitophagy.

## Data Availability

The datasets utilized in this study, including GSE194331 and GSE279876, are available for download from the GEO database (https://www.ncbi.nlm.nih.gov/geo/).
